# Impact of Partial Volume Correction on [^18^F]GE-180 PET Quantification in Subcortical Brain Regions of Patients with Corticobasal Syndrome

**DOI:** 10.3390/brainsci12020204

**Published:** 2022-01-31

**Authors:** Sebastian Schuster, Leonie Beyer, Carla Palleis, Stefanie Harris, Julia Schmitt, Endy Weidinger, Catharina Prix, Kai Bötzel, Adrian Danek, Boris-Stephan Rauchmann, Sophia Stöcklein, Simon Lindner, Marcus Unterrainer, Nathalie L. Albert, Lena M. Mittlmeier, Christian Wetzel, Rainer Rupprecht, Axel Rominger, Peter Bartenstein, Robert Perneczky, Johannes Levin, Günter U. Höglinger, Matthias Brendel, Franziska J. Dekorsy

**Affiliations:** 1 Department of Nuclear Medicine, University Hospital, Ludwig Maximilian University of Munich, 81377 Munich, Germany; Sebastian.Schuster@med.uni-muenchen.de (S.S.); Leonie.Beyer@med.uni-muenchen.de (L.B.); Stefanie.Harris@med.uni-muenchen.de (S.H.); Julia.Schmitt@med.uni-muenchen.de (J.S.); Simon.Lindner@med.uni-muenchen.de (S.L.); Nathalie.Albert@med.uni-muenchen.de (N.L.A.); Lena.Mittlmeier@med.uni-muenchen.de (L.M.M.); axel.rominger@insel.ch (A.R.); peter.bartenstein@med.uni-muenchen.de (P.B.); Matthias.Brendel@med.uni-muenchen.de (M.B.); 2 Department of Neurology, University Hospital of Munich, Ludwig Maximilian University of Munich, 81377 Munich, Germany; Carla.Palleis@med.uni-muenchen.de (C.P.); Endy.Weidinger@med.uni-muenchen.de (E.W.); catharina.prix@med.uni-muenchen.de (C.P.); Kai.Boetzel@med.uni-muenchen.de (K.B.); adrian.danek@med.uni-muenchen.de (A.D.); Johannes.Levin@med.uni-muenchen.de (J.L.); 3 Munich Cluster for Systems Neurology (SyNergy), 81377 Munich, Germany; Robert.Perneczky@med.uni-muenchen.de (R.P.); Hoeglinger.Guenter@mh-hannover.de (G.U.H.); 4 Department of Radiology, University Hospital of Munich, Ludwig Maximilian University of Munich, 81377 Munich, Germany; Boris.Rauchmann@med.uni-muenchen.de (B.-S.R.); sophia.stoecklein@med.uni-muenchen.de (S.S.); Marcus.Unterrainer@med.uni-muenchen.de (M.U.); 5 Department of Psychiatry and Psychotherapy, University Hospital, Ludwig Maximilian University of Munich, 81377 Munich, Germany; 6 Department of Psychiatry and Psychotherapy, University of Regensburg, 93053 Regensburg, Germany; Christian.Wetzel@klinik.uni-regensburg.de (C.W.); Rainer.Rupprecht@medbo.de (R.R.); 7 Department of Nuclear Medicine, University of Bern, Inselspital, CH-3010 Bern, Switzerland; 8 Metabolic Biochemistry, Biomedical Center (BMC), Faculty of Medicine, LMU Munich, 82152 Planegg, Germany; 9 German Center for Neurodegenerative Diseases (DZNE), 81377 Munich, Germany; 10 Ageing Epidemiology (AGE) Research Unit, School of Public Health, Imperial College, London SW7 2AZ, UK; 11 Department of Neurology, Hannover Medical School, 30625 Hannover, Germany

**Keywords:** corticobasal syndrome, partial volume effect, positron emission tomography, TSPO PET, [^18^F]GE-180, subcortical atrophy

## Abstract

Corticobasal syndrome (CBS) is a rare neurodegenerative condition characterized by four-repeat tau aggregation in the cortical and subcortical brain regions and accompanied by severe atrophy. The aim of this study was to evaluate partial volume effect correction (PVEC) in patients with CBS compared to a control cohort imaged with the 18-kDa translocator protein (TSPO) positron emission tomography (PET) tracer [^18^F]GE-180. Eighteen patients with CBS and 12 age- and sex-matched healthy controls underwent [^18^F]GE-180 PET. The cortical and subcortical regions were delineated by deep nuclei parcellation (DNP) of a 3D-T1 MRI. Region-specific subcortical volumes and standardized uptake values and ratios (SUV and SUVr) were extracted before and after region-based voxel-wise PVEC. Regional volumes were compared between patients with CBS and controls. The % group differences and effect sizes (CBS vs. controls) of uncorrected and PVE-corrected SUVr data were compared. Single-region positivity in patients with CBS was assessed by a >2 SD threshold vs. controls and compared between uncorrected and PVE-corrected data. Smaller regional volumes were detected in patients with CBS compared to controls in the right ventral striatum (*p* = 0.041), the left putamen (*p* = 0.005), the right putamen (*p* = 0.038) and the left pallidum (*p* = 0.015). After applying PVEC, the % group differences were distinctly higher, but the effect sizes of TSPO uptake were only slightly stronger due to the higher variance after PVEC. The single-region positivity of TSPO PET increased in patients with CBS after PVEC (100 vs. 83 regions). PVEC in the cortical and subcortical regions is valuable for TSPO imaging of patients with CBS, leading to the improved detection of elevated [^18^F]GE-180 uptake, although the effect sizes in the comparison against the controls did not improve strongly.

## 1. Introduction

Corticobasal syndrome (CBS) dominantly belongs to four-repeat (4R) tauopathies, which are characterized by the accumulation of hyperphosphorylated tau proteins in neurons and glial cells [1]. CBS is a rare brain disorder, clinically defined by extrapyramidal motor signs, including rigidity, akinesia, dystonia and myoclonus, and cortical signs such as apraxia, cortical sensory loss or alien limb phenomenon [2,3]. The mean survival time is estimated to be 5–8 years after symptom onset. Corticobasal degeneration is the distinct histopathological diagnosis behind the clinical presentation of the CBS symptoms [4]. The tau spread throughout the brain is driven by the release of toxic tau out of the cells into the extracellular space, followed by interactions and internalization into other cells [5,6,7,8]. Astrocytic pathology in corticobasal degeneration initially predominates in the frontoparietal and motor cortical areas and striatum, followed by other subcortical nuclei and, finally, the brainstem.

Neuroinflammation plays a pivotal role in neurodegenerative diseases and is characterized by the reactive morphology of glial cells, including both astrocytes and activated microglia, accompanied by elevated levels of proinflammatory molecules that are found in the brain regions affected by tau pathology [9,10]. Ongoing debates argue if neuroinflammation is the cause or effect of neurodegeneration. Microglia are the resident immune cells in the brain and have a nuanced role in neuroprotection. Under pathological conditions, microglia become activated and have the capacity to migrate, proliferate and efficiently phagocytose pathogens and cellular debris, including protein aggregates [10,11]. Thus, activated microglia may contribute to tau spreading by increasing its propagation and aggregation [12,13,14]. Additionally, the expression of proinflammatory molecules, e.g., IL-1β, TNF-α and IL-6, all feed into a cascade that leads to increases in tau hyperphosphorylation, reduction in synapse markers and neuronal loss [15].

The 18-kDa translocator protein (TSPO) is widely used as an imaging biomarker target of neuroinflammation. TSPO is dominantly overexpressed on activated microglia and can be imaged with radiotracers like [^18^F]GE-180 using positron emission tomography (PET). TSPO PET has been used for imaging neurodegenerative diseases [16], mostly in Alzheimer’s disease [17], β-amyloid mouse models [18] and less frequently in 4R-tauopathies, reporting pronounced subcortical neuroinflammation [19,20,21]. Due to cortical and subcortical atrophy in CBS, PET imaging suffers from partial volume effects (PVE), caused by limited spatial resolution of the scanner [22]. Previous studies with various radiotracers highlight the impact of partial volume effect correction (PVEC) to rescue the relative tracer signal and thereby reveal a more precise amount of the underlying target [22,23,24,25,26,27]. However, the value of PVEC for PET has mostly been explored for cortical atrophy but not for subcortical atrophy in CBS. A previous study by our research group indicated elevated TSPO PET binding in patients with CBS, fitting to predilection sites of this phenotype in comparison to the controls, but the effect sizes were moderate, and we did not observe any associations of TSPO PET binding and the parameters of disease severity [21]. Therefore, the aim of this study was to evaluate the use of subcortical PVEC for the assessment of TSPO PET in predefined subcortical target regions in patients with CBS and a matched control cohort to further clarify the sensitivity of 18-kDa translocator protein PET.

## 2. Materials and Methods

### 2.1. Study Design, Study Population and Clinical Assessment

TSPO PET imaging of the cohort has been reported previously by Palleis et al., and all CBS subjects with an available 3D T1 MRI were included in this PVEC study [21]. Eighteen patients with CBS (age 66.00 ± 7.46 years, 7 male) were recruited at the Department of Neurology, Ludwig Maximilians University (LMU) of Munich, together with age- and sex- matched controls without objective cognitive impairment or motor symptoms (*n* = 12; age 70.4 ± 7.5 years, 6 male). The neuropsychological tests PSP Rating Scale (PSP-RS), Montreal Cognitive Assessment (MoCA) and Schwab and England Activities of Daily Living (SEADL) served as the parameters for disease severity, severity of cognitive deficits and as the global score of functional ability, respectively. Detailed demographics and neuropsychological data of the included subjects are enclosed in Table 1. All participants provided informed written consent in accordance with the Declaration of Helsinki. The study was approved by the local ethics committee of LMU of Munich (ethics applications: 17-569 and 17-755 and their amendments).

### 2.2. TSPO PET Acquisition

All patients and controls were scanned using a Biograph 64 PET/CT scanner (Siemens, Erlangen, Germany); PET acquisition was followed by a low-dose CT serving for attenuation correction, as described previously [21]. Automated production of [^18^F]GE-180 was performed as previously specified [28]. In brief, three-dimensional image data were acquired 60–80 min after injection of approximately 180-MBq [^18^F]GE-180. PET was reconstructed with the TrueX algorithm (4 iterations, 16 subsets, all-pass, 336 × 336 pixel per slice (1.02 × 1.02 mm^2^)).

In addition to the TSPO PET, all included participants underwent an MRI scan at the Department of Radiology, University Hospital, LMU of Munich on a Siemens 3T Magnetom Skyra MR system (Siemens Healthineers, Erlangen, Germany). A 0.8-cm isovoxel high-resolution T1-weighted structural MRI sequence (repetition time (TR), 2060 ms; echo time (TE), 2.17 ms; flip angle (FA), 12 deg and field of view (FoV), 240 mm) was acquired.

### 2.3. Data Analysis and PVEC

The processing pipeline was performed using PMOD (V3.9, PMOD technologies, Zurich, Switzerland) and contained different steps. All steps were performed with the PNEURO toolbox. The PET images were cropped, and the MR images were cropped and denoised to prevent issues of large and inhomogeneous MRI fields of view (i.e., larger fields of view than the brain dimensions). Subsequently, all MR images were segmented into grey matter (gm), white matter (wm) and cerebrospinal fluid (CSF), followed by deep nuclei parcellation (DNP). The DNP determined 21 brain sections, based on the individual segmented MR image and the Hammers atlas base of the software [29]. Ten deep nuclei in both hemispheres were segmented: the ventral striatum, the caudate, the putamen, the pallidum and the thalamus, as well as 11 other regions of interests: the hippocampus, the amygdala, the cerebellar grey matter, the cerebellar white matter, the cortex, the white matter and cerebral spinal fluid (CSF). In the next step, the PET image and the segmented MR image were matched by a rigid co-registration using statistical parametric mapping algorithms (SPM). The individual T1-weighted MR image was co-registered nonlinearly to the MR atlas implemented in PMOD to match the parcellation of the brain with Hammers atlas regions of interests (ROIs) [29].

Finally, the brain sections were outlined, which allowed calculating region-based voxel-wise partial volume effect correction (PVEC) [22].

Various methods for PVEC are available. In this study, the region-based voxel-wise (RBV) PVE correction was used. Thomas et al. were able to show that the RBV method provides accurate results for PVEC compared also to other correction methods, especially for neurodegenerative diseases [22]. The advantage of RBV correction, compared to other methods, is the combination of the Geometric Transfer Matrix (GTM) method and voxel-wise correction. The GTM method parcellates the brain into nonoverlapping regions of interest (ROIs) using the anatomical information from the MR scan. It is assumed that the activities within the different ROIs are homogeneously distributed over their respective ROIs and represented by the mean value of the ROIs. The mean values affected by the partial volume effect (PVE) are determined and held as vectors. These vectors can then be used to create a matrix that is used to recover the actual activity of the multiple regions and correct for the spill-over between regions [30]. Ultimately, the GTM correction is a system consisting of linear equations.

When applying the RBV method, several steps are conducted. First, GTM correction is performed, resulting in an image with the correct values of the mean activity of the ROIs. These values are needed, as they are part of the correction term with which the PET image is multiplied in the next step. In the second step, a voxel-wise correction is performed by multiplying the uncorrected PET image by the correction term. This term consists of the GTM-corrected image from the first step and the point spread function (PSF) of the system. The result is an image in which the spill-over between the different regions has been corrected. In addition, the variability of the individual tissue types is taken into account by parceling the tissue types into smaller and more homogeneous groups.

The PVEC parameters included the following PET specifications: full-width half-maximum of X = 5.6 mm, Y = 5.6 mm and Z= 5.6 mm; TrueX algorithm (4 iterations, 16 subsets, all-pass, 336 × 336 pixel per slice (1.02 × 1.02 mm^2^)). For semiquantitative analyses, PET standard uptake values (SUVs) were extracted from all subcortical target regions (21 regions, including the cerebellum and cortex) for corrected and uncorrected images. The mean SUV and standard uptake value ratios (SUVr) relative to the anterior temporal lobe were calculated for each volume of interest (VOI) in all subjects. Palleis et al. previously defined the anterior lateral temporal lobe as suitable pseudo-reference tissue, as the SUVr showed no significant difference between patients with 4R tauopathies and controls [21]. The volumes of the different regions were determined from the individual MR images by PMOD software.

### 2.4. rs6971 Single Nucleotide Polymorphism

TSPO PET tracers are sensitive to a single-nucleotide polymorphism (rs6971) that replaces alanine with threonine (Ala147Thr), resulting in three patterns of binding affinity: high-affinity binders (HABs), medium-affinity binders (MABs) and low-affinity binders (LABs), depending on the homozygosity or heterozygosity of the allele. The binding affinity was evaluated by genotyping for the rs6971 polymorphism at the Department of Psychiatry of the University Hospital Regensburg, as previously described, and each participant was classified as either LAB, MAB or HAB [31]. LABs were excluded in this study, due to known significantly lower [^18^F]GE-180 binding in controls when compared to MABs and HABs, whereas there was no significant difference between MABs and HABs [32].

### 2.5. Statistical Analysis

For the statistical processing of the data and illustration of the results, Excel (Microsoft, Redmond, WA, USA) and SPSS Version 27 (IBM, Ehningen, Germany) were used.

A chi-square test was used to ensure homogeneity between the two groups in terms of sex and rs6971 polymorphism. Age was compared between the study groups (CBS and healthy controls (HC)) by a 1-way analysis of variance. Volumes of the predefined subcortical and cortical target regions were compared between patients with CBS and the controls using a Wilcoxon Mann–Whitney test.

SUV and SUVr before and after PVEC were compared between both groups by a nonparametric Wilcoxon Mann–Whitney test. Further, a paired *t*-test was executed to show the significant differences of the mean SUV and SUVr before and after correction for the partial volume effect. Statistical significance was defined as two-tailed *p*-values < 0.05. Percentage differences before and after PVE correction between the single subcortical regions were calculated. In addition, for each regional difference, the effect size was determined by Pearson correlation coefficient. For region-based classification, regional SUVr ≥ mean value + 2 standard deviations of the controls were defined as positive.

Partial correlation was performed to measure the degree of association between SUVr from the subcortical regions and clinical data (Progressive Supranuclear Palsy rating scale (PSP rating scale), disease duration, Montreal Cognitive Assessment (MoCA) and Schwab and England Activities of Daily Living scale (SEADL)) adjusted for age, gender and the TSPO-binding polymorphism.

## 3. Results

### 3.1. Demographics and Clinical Data

The eighteen included patients with CBS did not significantly differ from the controls in age (66.00 ± 7.46 years vs. 70.42 ± 7.45 years, *p* = 0.14) and gender (*p* = 0.65). The rs6971 polymorphism had a shift towards a higher proportion of HABs in patients with CBS compared to the controls (*p* = 0.01); though MABs and HABs have been proven to not differ significantly in terms of [^18^F]GE-180 binding [32]. Low-affinity binding subjects for the rs6971 single-nucleotide polymorphism were excluded, as described above. The mean disease duration of patients with CBS was 30.83 ± 19.47 months. Patients with CBS showed an impaired PSP rating scale and SEADL and scored lower for cognitive performance testing by MoCA (23.39 ± 4.36) compared to the controls (29.00 ± 1.00; *p* < 0.001) (Table 1).

### 3.2. Subcortical Brain Regions in CBS Patients Show Volume Loss When Compared to Controls

The MRI volumes, assessed by individual T1-weighted images, differed in all subcortical regions in patients with CBS compared to the controls as a sign of severe atrophy. Significantly smaller volumes were detected in the following subcortical regions: the right ventral striatum (*p* = 0.041), the left putamen (*p* = 0.005), the right putamen (*p* = 0.038) and the left pallidum (*p* = 0.015). The overall cortical volumes of both hemispheres were reduced in patients with CBS when compared to the controls (right *p* = 0.057, left *p* = 0.047). All regions studied are shown in Table 2.

The anterior temporal lobe, which was used as a predefined reference region, did not show a significant volume change in patients with CBS compared to the controls (left: *p* = 0.398, right: *p* = 0.353). The cerebellum, as a possible reference region for neurodegenerative imaging, does not present volume differences between patients with CBS and the controls (cerebellar white matter: *p* = 0.204, cerebellar grey matter: *p* = 0.866).

### 3.3. Region-Based PVEC in TSPO PET in Subcortical Brain Regions

Standardized uptake values (SUVs) were assessed in patients with CBS and in the controls before and after PVEC. The mean SUVs over all the predefined subcortical regions of interest were slightly higher in patients with CBS before (SUVcontrols 0.81 ± 0.21, SUVcbs 0.83 ± 0.17; *p* = 0.716) and after PVEC (mean SUVcontrols 0.79 ± 0.22, mean SUVcbs 0.84 ± 0.20; *p* = 0.603) (Table 3).

The PVEC effect in the anterior temporal lobe was slightly lower in patients with CBS compared to the controls (left: *p* = 0.176, right: *p* = 0.236). In the cerebellum, the PVEC effect did not differ in patients with CBS compared to the controls (*p* = 0.735).

Considering all regions of interest (ROIs), the change in SUV after PVEC in patients with CBS was comparable to those in the controls (*p* = 0.603). On a regional level, though, the change of SUV after PVEC was significantly higher in patients with CBS compared to the controls in the right amygdala (*p* = 0.022), the right thalamus (*p* = 0.047) and the left thalamus (*p* = 0.014), and a trend towards a significant level was reached in the left putamen (*p* = 0.057) and the left pallidum (*p* = 0.075).

**Figure 1:**
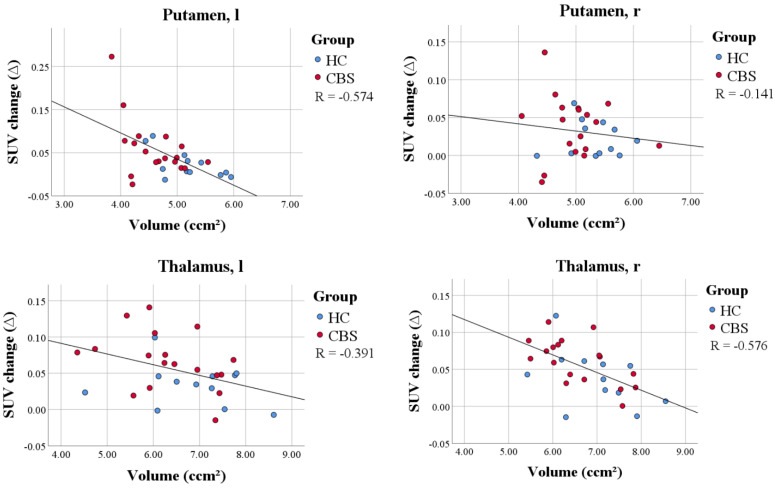
Correlation plot of the volume and PVEC-induced change of SUV within the whole study group in the left putamen, right putamen, left thalamus and right thalamus. Patients with CBS (red) showed higher differences in SUV after PVEC and smaller volumes compared to healthy controls (blue). Smaller volumes were associated with a higher PVEC-induced change of SUV, which validated the performance of the PVEC approach in the subcortical brain regions.

In the next step, we correlated the PVEC-induced change of SUV with the volume in the whole study cohort (patients with CBS and the controls). We hypothesized higher changes in SUV after PVEC in correlation with more severe individual atrophy. A significant correlation of the change of the SUV and volume was found in the left putamen (*p* = 0.005, 95% CI: −0.806 to −0.101), the left thalamus (*p* = 0.045, 95% CI: −0.671 to −0.002) and the right thalamus (*p* < 0.001, 95% CI: −0.841 to −0.247), fitting to the topology of the volume loss in CBS [33] (Figure 1). Regarding the reference region, neither the anterior temporal lobe or the cerebellum showed a significant correlation between the PVEC-induced change in the SUV and the volume.

### 3.4. Impact of PVEC on SUVr Differences and Effect Sizes for the Comparison of CBS and Controls

The percentage difference in the mean SUVr between CBS patients and HC increased significantly in the subcortical regions after applying PVEC (18.6 ± 0.1% vs. 11.1 ± 0.1%, *p* = 0.021; Table 4). After PVEC, the largest change in percentage difference of the mean SUVr between patients with CBS and HC was observed in the left caudate with 16.9% and right amygdala with 10.7%. The number of subcortical regions with a strong effect size increased slightly after PVEC, but the effect sizes in regions with the highest TSPO PET increases of the patients with CBS vs. the controls did not improve significantly after PVEC (Table 4).

### 3.5. Single Region Positivity of TSPO PET before and after PVEC

**Figure 2:**
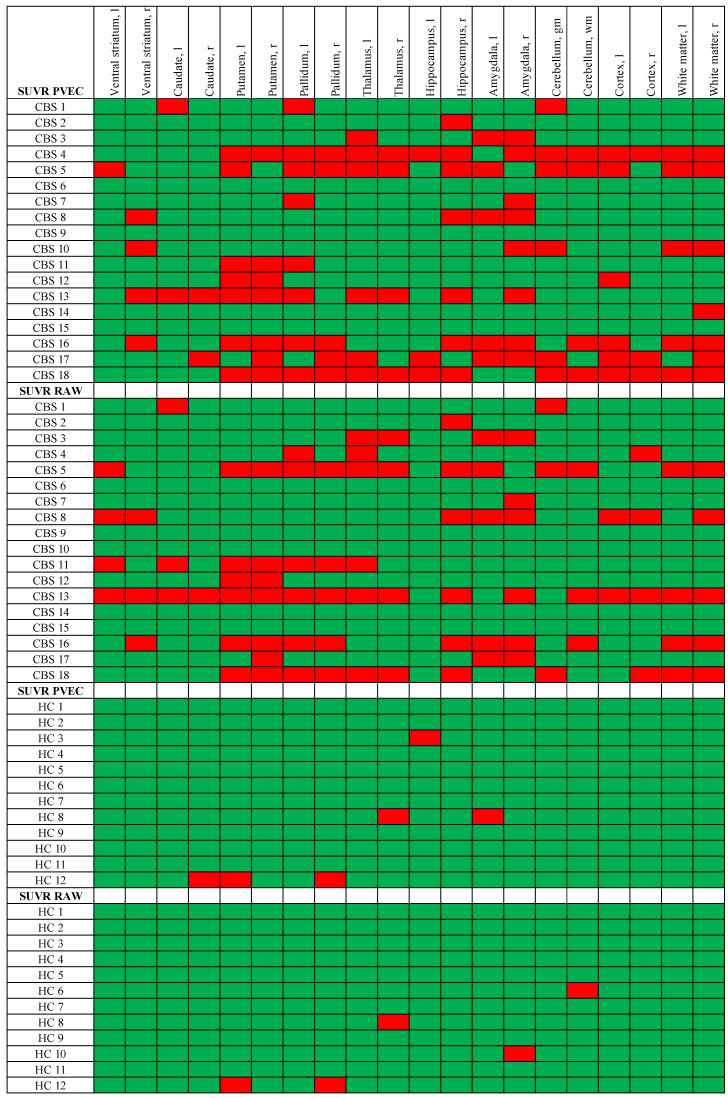
Classifier of single-region positivity for patients with CBS by TSPO PET. Semiquantitative classification (red = positive, green = negative) of the target regions for CBS was performed by applying the mean value + 2 standard deviations (SD) threshold obtained from the healthy controls.

On the single-region level, positivity in patients with CBS and HC was assessed by a >2 SD threshold and compared between uncorrected and PVE-corrected data in both groups. The single-region positivity in CBS increased after PVEC, resulting in 100 regions after PVEC compared to 83 regions before correction. In the HC group, the single-region positivity increased slightly after PVEC, resulting in six regions after PVEC compared to five regions before the correction (Figure 2).

### 3.6. PVEC Influence on the Association of Disease Parameter with TSPO Labeling

**Figure 3:**
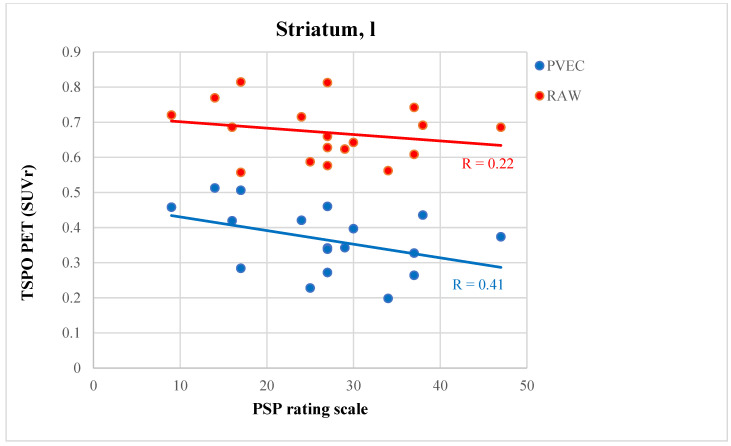
Correlation plot of the PSP rating scale and TSPO PET SUVr in the left striatum before (red) and after (blue) PVEC.

SUVr of the predefined regions were correlated with disease-related parameters before and after PVEC to assess the influence of atrophy on associations between TSPO PET and clinical severity. TSPO labeling in the subcortical regions did not correlate with the disease duration either before or after PVEC (before PVEC *p* = 0.564, after PVEC *p* = 0.704), MoCA (before PVEC *p* = 0.357, after PVEC *p* = 0.524), SEADL (before PVEC *p* = 0.421, after PVEC *p* = 0.325) or PSP-RS (before PVEC *p* = 0.529, after PVEC *p* = 0.598). In a few regions, after PVEC, the correlation of the PSP rating scale and TSPO PET SUVr increased slightly after PVEC but still did not indicate significance. The most pronounced increase after PVEC was observed in the left caudate (before PVEC *p* = 0.449, r = 0.22, after PVEC *p* = 0.205, r = 0.41; Figure 3).

## 4. Discussion

In this study, the effect of PVEC in the subcortical regions was evaluated in patients with CBS imaged with the TSPO radiotracer [^18^F]GE-180. Atrophy in patients with CBS predominantly involved the subcortical regions, including the thalamus, pallidum and putamen. In these atrophic subcortical regions of patients with CBS, we observed stronger PVEC-induced changes of SUV when compared to the controls, thus proving the clinical usefulness of the PVEC method for subcortical brain compartments. PVEC also increased the difference of the tracer uptake for the comparison of patients with CBS and the controls, but a parallel increase of the group-based variance did not result in stronger effect sizes for the group comparisons of patients with CBS vs. the controls.

In the disease course of neurodegenerative disorders, progressive atrophy of the affected brain regions impacts the quantification of molecular imaging using PET due to PVE. Due to the low resolution of PET, PVE leads to a substantial reduction of the measured PET tracer signal. While PVE can lead to false-positive findings of reduced FDG uptake in neurodegenerative diseases [34], tracers aiming to measure a target elevation in neurodegenerative diseases, such as β-amyloid [23,24], tau [35,36] or TSPO ligands, can be compromised in their sensitivity. Partial volume effect correction has extensively been studied for cortical atrophy in neurodegenerative diseases for various radiotracers [22]. However, severe atrophy in patients with CBS also affects the small subcortical regions, mostly the basal ganglia [37,38], and PVEC in the subcortical regions has not yet been studied and validated extensively in neurodegenerative diseases. As hypothesized, volume reduction was significantly detectable in patients with CBS compared to the controls primarily in the subcortical regions and most notably in the right ventral striatum, the left putamen, the right putamen and the left pallidum, matching the known most-affected subcortical regions by CBS [33,39,40]. Across all subcortical regions, the change of SUV before and after PVEC in patients with CBS compared to those in the controls was not significantly different. Interestingly though, on the regional level, the change of SUV (before and after PVEC) was significantly higher in patients with CBS compared to the controls. This can be explained due to the proceeding and CBS-specific atrophy in those subcortical regions (amygdala, thalamus, putamen and pallidum).

To validate the used algorithm, assuming the higher the atrophy, the higher the change in SUV after PVEC, a correlation of the change of the SUV with the volume was performed. The most significant correlation of the change in SUV with the volume was found in the left putamen, the left thalamus and the right thalamus, which are the most affected regions by CBS. This may confirm the importance of PVEC in molecular imaging of the subcortical regions, at least in patients with CBS.

Neuroinflammation or, more specifically, proinflammatory activation of the neuroimmune cells’ microglia and astrocytes is thought to be a contributor to the pathological process of neurodegenerative diseases. Several studies have focused on the investigation of activated microglia by PET in various neurodegenerative diseases by several PET ligands [19,41,42,43,44,45]. Among others, our group has recently shown that elevated TSPO labeling in patients with CBS corresponds to the expected topology, and we demonstrated that [^18^F]GE-180 can detect neuroinflammation in 4R tauopathies, including CBS [21]. Here, we were able to confirm that elevated microglial activation mainly occurs in disease-specific subcortical regions of CBS, but we questioned if subcortical atrophy counterbalances even higher effects. The percentage difference in SUV between patients with CBS and the healthy controls before PVEC in our subset and in the full cohort of Palleis et al. were comparable with 20% and 24%, respectively. Thus, our subset (*n* = 18) of patients with an available MRI is a representative cohort. However, it should be noted that the methodological variance is naturally higher due to the smaller group.

After PVEC, we were able to detect higher percentage differences of TSPO PET binding in patients with CBS vs. the controls. However, the corresponding effect size was only slightly improved in a few regions after PVEC. This can be explained by increasing the variance of TSPO PET quantification when using PVEC, as already shown for other tracers [23,24]. Our single-region assessment indicated that at least a few atrophic cases could be detected better when using PVEC.

In this regard, our previous study did not indicate any correlation of the disease parameters with TSPO uptake [21]. Thus, we questioned if atrophy, which aggravates during the disease course and could lead to a PVE-associated false low signal in the late stage, may cause this missing correlation. However, the correlation of disease parameters, including disease duration the PSP rating scale and SEADL with the TSPO uptake, did not increase after PVEC, suggesting that there is indeed no linear relationship between disease severity and activated microglia.

Among the limitations of our study, we note missing autopsy validation of the studied, clinically diagnosed patients with CBS, though the specificity of a clinical 4R tauopathy is very high. We acknowledge as another limitation of this study the small sample size, which is due to the rarity of the disease and the novel tracer [^18^F]GE-180, which results in rather low statistical power. Our results should be interpreted as the first direction and proof of concept for PVEC in subcortical regions. A third limitation might be the relatively low uptake of [^18^F]GE-180, which has been shown to be specific, though [21,46,47].

## 5. Conclusions

In conclusion, PVEC to correct for subcortical atrophy leads to improved [^18^F]GE-180 signal detection in patients with CBS, which shows the value of also using PVEC for subcortical molecular imaging.

## Figures and Tables

**Table 1:** Demographics of the two study groups.

	CBS	HC
*n*	18	12
Age (y)	66.00 ± 7.46	70.42 ± 7.45
Sex	♀ 11/♂ 7	♀ 6/♂ 6
rs6971	HAB: 14/MAB: 4	HAB: 4/MAB: 8
PSP rating scale	26.78 ± 9.45	n.a.
Disease duration (m)	30.83 ± 19.47	n.a.
MoCA	23.39 ± 4.36	29.00 ± 1.00
SEADL	63.89 ± 13.80	n.a.

**Table 2:** Volumes of the predefined regions of interest in both groups with the significant *p*-values highlighted in bold.

Region	Mean vol. CBS (ccm ± SD)	Mean vol. HC (ccm ± SD)	*p*-Value
Ventral striatum, l	0.47 ± 0.06	0.48 ± 0.05	0.498
Ventral striatum, r	0.46 ± 0.04	0.49 ± 0.06	**0.041**
Caudate, l	4.11 ± 0.64	4.35 ± 0.69	0.374
Caudate, r	4.29 ± 0.60	4.77 ± 0.60	0.086
Putamen, l	4.61 ± 0.47	5.19 ± 0.48	**0.005**
Putamen, r	4.97 ± 0.53	5.32 ± 0.44	**0.038**
Pallidum, l	1.52 ± 0.22	1.79 ± 0.27	**0.015**
Pallidum, r	1.65 ± 0.15	1.81 ± 0.21	0.065
Thalamus, l	6.34 ± 0.97	6.87 ± 1.04	0.133
Thalamus, r	6.57 ± 0.78	6.99 ± 0.85	0.175
Hippocampus, l	3.95 ± 0.49	3.89 ± 0.32	0.703
Hippocampus, r	3.86 ± 0.35	3.97 ± 0.36	0.397
Amygdala, l	1.66 ± 0.19	1.71 ± 0.20	0.611
Amygdala, r	1.78 ± 0.23	1.75 ± 0.27	0.882
Cerebellum, gm	102.50 ± 23.27	101.76 ± 27.23	0.866
Cerebellum, wm	36.24 ± 8.47	37.42 ± 10.83	0.204
Cortex, l	298.70 ± 22.08	325.59 ± 36.22	0.057
Cortex, r	302.01 ± 25.64	318.41 ± 17.91	**0.047**
White matter, l	237.06 ± 23.70	248.52 ± 25.66	0.374
White matter, r	239.64 ± 20.07	248.18 ± 22.34	0.290
Anterior temporal lobe, l	4.06 ± 1.53	3.92 ± 1.54	0.398
Anterior temporal lobe, r	4.14 ± 1.54	3.99 ± 1.53	0.353

**Table 3:** Mean SUVs before and after PVEC in patients with CBS and healthy controls and the corresponding significances.

	CBS			HC		
Region	Mean SUV before PVEC ± SD	Mean SUV after PVEC ± SD	*p*	Mean SUV before PVEC ± SD	Mean SUV after PVEC ± SD	*p*
Ventral striatum, l	0.888 ± 0.20	1.022 ± 0.34	0.177	0.904 ± 0.21	1.112 ± 0.31	0.002
Ventral striatum, r	0.919 ± 0.20	1.107 ± 0.37	0.149	0.948 ± 0.22	1.185 ± 0.35	0.002
Caudate, l	0.594 ± 0.12	0.445 ± 0.12	0.001	0.582 ± 0.13	0.387 ± 0.12	0.002
Caudate, r	0.584 ± 0.13	0.432 ± 0.18	0.017	0.619 ± 0.16	0.465 ± 0.16	0.002
Putamen, l	0.875 ± 0.19	0.934 ± 0.23	0.463	0.805 ± 0.19	0.828 ± 0.20	0.028
Putamen, r	0.849 ± 0.17	0.886 ± 0.20	0.287	0.810 ± 0.20	0.832 ± 0.21	0.010
Pallidum, l	0.890 ± 0.18	0.979 ± 0.21	0.084	0.807 ± 0.20	0.862 ± 0.23	0.002
Pallidum, r	0.878 ± 0.17	0.969 ± 0.20	0.068	0.818 ± 0.19	0.883 ± 0.21	0.002
Thalamus, l	0.955 ± 0.19	1.021 ± 0.22	0.149	0.907 ± 0.23	0.940 ± 0.25	0.008
Thalamus, r	0.938 ± 0.17	0.999 ± 0.19	0.149	0.916 ± 0.25	0.954 ± 0.28	0.008
Hippocampus, l	0.834 ± 0.16	0.852 ± 0.19	0.619	0.895 ± 0.23	0.937 ± 0.27	0.010
Hippocampus, r	0.833 ± 0.16	0.855 ± 0.19	0.723	0.845 ± 0.19	0.848 ± 0.20	0.875
Amygdala, l	0.835 ± 0.18	0.815 ± 0.22	0.831	0.858 ± 0.22	0.811 ± 0.23	0.006
Amygdala, r	0.846 ± 0.17	0.834 ± 0.21	0.943	0.848 ± 0.21	0.785 ± 0.21	0.002
Cerebellum, gm	0.880 ± 0.15	1.002 ± 0.19	0.015	0.921 ± 0.24	1.033 ± 0.27	0.002
Cerebellum, wm	0.826 ± 0.16	0.800 ± 0.17	0.653	0.852 ± 0.20	0.817 ± 0.19	0.008
Cortex, l	0.860 ± 0.16	1.049 ± 0.21	0.006	0.903 ± 0.22	1.083 ± 0.27	0.002
Cortex, r	0.871 ± 0.16	1.073 ± 0.21	0.006	0.927 ± 0.23	1.129 ± 0.28	0.002
White matter, l	0.760 ± 0.14	0.664 ± 0.13	0.062	0.765 ± 0.17	0.648 ± 0.14	0.002
White matter, r	0.759 ± 0.14	0.659 ± 0.13	0.084	0.771 ± 0.18	0.643 ± 0.14	0.002
Anterior temporal lobe, l	0.906 ± 0.16	1.261 ± 0.21	0.001	0.998 ± 0.24	1.460 ± 0.36	0.002
Anterior temporal lobe, r	0.870 ± 0.16	1.193 ± 0.23	0.001	0.938 ± 0.24	1.320 ± 0.41	0.002

**Table 4:** Percentage differences in the mean SUVr at the group level before and after PVEC in the subcortical regions for patients with CBS and the healthy controls. The corresponding effect size was strong (r > 0.5, highlighted in bold) in more subcortical regions after PVEC than prior to PVEC. ∆ (%) = change in SUV differences.

Regions	Before PVEC—CBS vs. HC (%)	r	After PVEC—CBS vs. HC (%)	r	∆ (%)
Ventral striatum, l	5.88	0.2	0.72	0.1	−5.16
Ventral striatum, r	5.72	0.2	5.73	0.1	0.01
Caudate nucl., l	10.44	0.3	27.32	0.4	16.88
Caudate nucl., r	2.67	0.1	5.56	0.1	2.89
Putamen, l	18.4	**0.5**	27.25	**0.5**	8.85
Putamen, r	14.14	**0.5**	19.99	**0.5**	5.85
Pallidum, l	20.75	**0.6**	29.38	**0.5**	8.63
Pallidum, r	16.57	**0.5**	22.8	**0.5**	6.23
Thalamus, l	14.81	**0.6**	22.8	**0.5**	7.99
Thalamus, r	12.37	**0.5**	19.1	**0.5**	6.73
Hippocampus, l	1.6	0.1	3.26	0.2	1.66
Hippocampus, r	6.83	0.4	13.01	**0.5**	6.18
Amygdala, l	6.03	0.2	13.08	0.2	7.05
Amygdala, r	8.44	0.4	19.09	**0.5**	10.65

## Data Availability

The data presented in this study are available on request from the corresponding author.

## References

[1] Ferrer I., López-González I., Carmona M., Arregui L., Dalfó E., Torrejón-Escribano B., Diehl R., Kovacs G.G. (2014). Glial and neuronal tau pathology in tauopathies: Characterization of disease-specific phenotypes and tau pathology progression. J. Neuropathol. Exp. Neurol..

[2] Chahine L.M., Rebeiz T., Rebeiz J.J., Grossman M., Gross R.G. (2014). Corticobasal syndrome: Five new things. Neurol. Clin. Pract..

[3] Armstrong M.J., Litvan I., Lang A.E., Bak T.H., Bhatia K.P., Borroni B., Boxer A.L., Dickson D.W., Grossman M., Hallett M. (2013). Criteria for the diagnosis of corticobasal degeneration. Neurology.

[4] Kouri N., Whitwell J.L., Josephs K.A., Rademakers R., Dickson D.W. (2011). Corticobasal degeneration: A pathologically distinct 4R tauopathy. Nat. Rev. Neurol..

[5] Simón D., García-García E., Royo F., Falcón-Pérez J.M., Avila J. (2012). Proteostasis of tau. Tau overexpression results in its secretion via membrane vesicles. FEBS Lett..

[6] Pooler A.M., Phillips E.C., Lau D.H., Noble W., Hanger D.P. (2013). Physiological release of endogenous tau is stimulated by neuronal activity. EMBO Rep..

[7] Gómez-Ramos A., Díaz-Hernández M., Cuadros R., Hernández F., Avila J. (2006). Extracellular tau is toxic to neuronal cells. FEBS Lett..

[8] Calafate S., Buist A., Miskiewicz K., Vijayan V., Daneels G., de Strooper B., de Wit J., Verstreken P., Moechars D. (2015). Synaptic Contacts Enhance Cell-to-Cell Tau Pathology Propagation. Cell Rep..

[9] Ransohoff R.M. (2016). How neuroinflammation contributes to neurodegeneration. Science.

[10] Leyns C.E.G., Holtzman D.M. (2017). Glial contributions to neurodegeneration in tauopathies. Mol. Neurodegener..

[11] Kettenmann H., Hanisch U.K., Noda M., Verkhratsky A. (2011). Physiology of microglia. Physiol. Rev..

[12] Kahlson M.A., Colodner K.J. (2015). Glial Tau Pathology in Tauopathies: Functional Consequences. J. Exp. Neurosci..

[13] Ising C., Venegas C., Zhang S., Scheiblich H., Schmidt S.V., Vieira-Saecker A., Schwartz S., Albasset S., McManus R.M., Tejera D. (2019). NLRP3 inflammasome activation drives tau pathology. Nature.

[14] Asai H., Ikezu S., Tsunoda S., Medalla M., Luebke J., Haydar T., Wolozin B., Butovsky O., Kügler S., Ikezu T. (2015). Depletion of microglia and inhibition of exosome synthesis halt tau propagation. Nat. Neurosci..

[15] von Bernhardi R., Eugenín-von Bernhardi L., Eugenín J. (2015). Microglial cell dysregulation in brain aging and neurodegeneration. Front. Aging Neurosci..

[16] Stefaniak J., O’Brien J. (2016). Imaging of neuroinflammation in dementia: A review. J. Neurol. Neurosurg. Psychiatry.

[17] Liu B., Le K.X., Park M.A., Wang S., Belanger A.P., Dubey S., Frost J.L., Holton P., Reiser V., Jones P.A. (2015). In Vivo Detection of Age- and Disease-Related Increases in Neuroinflammation by ^18^F-GE180 TSPO MicroPET Imaging in Wild-Type and Alzheimer’s Transgenic Mice. J. Neurosci..

[18] Parhizkar S., Arzberger T., Brendel M., Kleinberger G., Deussing M., Focke C., Nuscher B., Xiong M., Ghasemigharagoz A., Katzmarski N. (2019). Loss of TREM2 function increases amyloid seeding but reduces plaque-associated ApoE. Nat. Neurosci..

[19] Gerhard A., Trender-Gerhard I., Turkheimer F., Quinn N.P., Bhatia K.P., Brooks D.J. (2006). In vivo imaging of microglial activation with [^11^C](R)-PK11195 PET in progressive supranuclear palsy. Mov. Disord..

[20] Passamonti L., Rodríguez P.V., Hong Y.T., Allinson K.S.J., Bevan-Jones W.R., Williamson D., Jones P.S., Arnold R., Borchert R.J., Surendranathan A. (2018). [(^11^)C]PK11195 binding in Alzheimer disease and progressive supranuclear palsy. Neurology.

[21] Palleis C., Sauerbeck J., Beyer L., Harris S., Schmitt J., Morenas-Rodriguez E., Finze A., Nitschmann A., Ruch-Rubinstein F., Eckenweber F. (2021). In Vivo Assessment of Neuroinflammation in 4-Repeat Tauopathies. Mov. Disord..

[22] Thomas B.A., Erlandsson K., Modat M., Thurfjell L., Vandenberghe R., Ourselin S., Hutton B.F. (2011). The importance of appropriate partial volume correction for PET quantification in Alzheimer’s disease. Eur. J. Nucl. Med. Mol. Imaging.

[23] Brendel M., Delker A., Rötzer C., Böning G., Carlsen J., Cyran C., Mille E., Gildehaus F.J., Cumming P., Baumann K. (2014). Impact of partial volume effect correction on cerebral β-amyloid imaging in APP-Swe mice using [(^18^)F]-florbetaben PET. Neuroimage.

[24] Brendel M., Högenauer M., Delker A., Sauerbeck J., Bartenstein P., Seibyl J., Rominger A. (2015). Improved longitudinal [(^18^)F]-AV45 amyloid PET by white matter reference and VOI-based partial volume effect correction. Neuroimage.

[25] Brendel M., Reinisch V., Kalinowski E., Levin J., Delker A., Därr S., Pogarell O., Förster S., Bartenstein P., Rominger A. (2016). Hypometabolism in Brain of Cognitively Normal Patients with Depressive Symptoms is Accompanied by Atrophy-Related Partial Volume Effects. Curr. Alzheimer Res..

[26] Rullmann M., McLeod A., Grothe M.J., Sabri O., Barthel H. (2020). Reshaping the Amyloid Buildup Curve in Alzheimer Disease? Partial-Volume Effect Correction of Longitudinal Amyloid PET Data. J. Nucl. Med..

[27] Rullmann M., Dukart J., Hoffmann K.T., Luthardt J., Tiepolt S., Patt M., Gertz H.J., Schroeter M.L., Seibyl J., Schulz-Schaeffer W.J. (2016). Partial-Volume Effect Correction Improves Quantitative Analysis of ^18^F-Florbetaben β-Amyloid PET Scans. J. Nucl. Med..

[28] Wickstrøm T., Clarke A., Gausemel I., Horn E., Jørgensen K., Khan I., Mantzilas D., Rajanayagam T., In’t Veld D.J., Trigg W. (2014). The development of an automated and GMP compliant FASTlab™ Synthesis of [^18^F] GE-180; a radiotracer for imaging translocator protein (TSPO). J. Label. Compd. Radiopharm..

[29] Hammers A., Allom R., Koepp M.J., Free S.L., Myers R., Lemieux L., Mitchell T.N., Brooks D.J., Duncan J.S. (2003). Three-dimensional maximum probability atlas of the human brain, with particular reference to the temporal lobe. Hum. Brain Mapp..

[30] Rousset O.G., Ma Y., Evans A.C. (1998). Correction for partial volume effects in PET: Principle and validation. J. Nucl. Med..

[31] Albert N.L., Unterrainer M., Fleischmann D., Lindner S., Vettermann F., Brunegraf A., Vomacka L., Brendel M., Wenter V., Wetzel C. (2017). TSPO PET for glioma imaging using the novel ligand 18 F-GE-180: First results in patients with glioblastoma. Eur. J. Nucl. Med. Mol. Imaging.

[32] Vettermann F.J., Harris S., Schmitt J., Unterrainer M., Lindner S., Rauchmann B.S., Palleis C., Weidinger E., Beyer L., Eckenweber F. (2021). Impact of TSPO Receptor Polymorphism on [(^18^)F]GE-180 Binding in Healthy Brain and Pseudo-Reference Regions of Neurooncological and Neurodegenerative Disorders. Life.

[33] Rohrer J.D., Lashley T., Schott J.M., Warren J.E., Mead S., Isaacs A.M., Beck J., Hardy J., de Silva R., Warrington E. (2011). Clinical and neuroanatomical signatures of tissue pathology in frontotemporal lobar degeneration. Brain.

[34] Ewers M., Brendel M., Rizk-Jackson A., Rominger A., Bartenstein P., Schuff N., Weiner M.W. (2014). Reduced FDG-PET brain metabolism and executive function predict clinical progression in elderly healthy subjects. Neuroimage Clin..

[35] Schöll M., Lockhart S.N., Schonhaut D.R., O’Neil J.P., Janabi M., Ossenkoppele R., Baker S.L., Vogel J.W., Faria J., Schwimmer H.D. (2016). PET Imaging of Tau Deposition in the Aging Human Brain. Neuron.

[36] Wang L., Benzinger T.L., Su Y., Christensen J., Friedrichsen K., Aldea P., McConathy J., Cairns N.J., Fagan A.M., Morris J.C. (2016). Evaluation of Tau Imaging in Staging Alzheimer Disease and Revealing Interactions Between β-Amyloid and Tauopathy. JAMA Neurol..

[37] Parmera J.B., de Almeida I.J., de Oliveira M.C.B., Silagi M.L., de Godoi Carneiro C., Studart-Neto A., Ono C.R., Reis Barbosa E., Nitrini R., Buchpiguel C.A. (2021). Metabolic and Structural Signatures of Speech and Language Impairment in Corticobasal Syndrome: A Multimodal PET/MRI Study. Front. Neurol..

[38] Boxer A.L., Geschwind M.D., Belfor N., Gorno-Tempini M.L., Schauer G.F., Miller B.L., Weiner M.W., Rosen H.J. (2006). Patterns of brain atrophy that differentiate corticobasal degeneration syndrome from progressive supranuclear palsy. Arch. Neurol..

[39] Tokumaru A.M., O’Uchi T., Kuru Y., Maki T., Murayama S., Horichi Y. (1996). Corticobasal degeneration: MR with histopathologic comparison. AJNR Am. J. Neuroradiol..

[40] Franceschi A.M., Clifton M., Naser-Tavakolian K., Ahmed O., Cruciata G., Bangiyev L., Clouston S., Franceschi D. (2021). ((^18^)F)-Fluorodeoxyglucose positron emission tomography/magnetic resonance imaging assessment of hypometabolism patterns in clinical phenotypes of suspected corticobasal degeneration. World J. Nucl. Med..

[41] Tournier B.B., Tsartsalis S., Ceyzériat K., Garibotto V., Millet P. (2020). In Vivo TSPO Signal and Neuroinflammation in Alzheimer’s Disease. Cells.

[42] Lagarde J., Sarazin M., Bottlaender M. (2018). In vivo PET imaging of neuroinflammation in Alzheimer’s disease. J. Neural Transm..

[43] Schain M., Kreisl W.C. (2017). Neuroinflammation in Neurodegenerative Disorders-a Review. Curr. Neurol. Neurosci. Rep..

[44] Sacher C., Blume T., Beyer L., Peters F., Eckenweber F., Sgobio C., Deussing M., Albert N.L., Unterrainer M., Lindner S. (2019). Longitudinal PET Monitoring of Amyloidosis and Microglial Activation in a Second-Generation Amyloid-β Mouse Model. J. Nucl. Med..

[45] Brendel M., Kleinberger G., Probst F., Jaworska A., Overhoff F., Blume T., Albert N.L., Carlsen J., Lindner S., Gildehaus F.J. (2017). Increase of TREM2 during Aging of an Alzheimer’s Disease Mouse Model Is Paralleled by Microglial Activation and Amyloidosis. Front. Aging Neurosci..

[46] Deussing M., Blume T., Vomacka L., Mahler C., Focke C., Todica A., Unterrainer M., Albert N.L., Lindner S., von Ungern-Sternberg B. (2018). Data on specificity of [(^18^)F]GE180 uptake for TSPO expression in rodent brain and myocardium. Data Brief.

[47] Sridharan S., Raffel J., Nandoskar A., Record C., Brooks D.J., Owen D., Sharp D., Muraro P.A., Gunn R., Nicholas R. (2019). Confirmation of specific binding of the 18-kDa translocator protein (TSPO) radioligand [^18^F] GE-180: A blocking study using XBD173 in multiple sclerosis normal appearing white and grey matter. Mol. Imaging Biol..

